# *Madurella mycetomatis* causing eumycetoma medical treatment: The challenges and prospects

**DOI:** 10.1371/journal.pntd.0008307

**Published:** 2020-08-27

**Authors:** Lamis Y. M. Elkheir, Rayan Haroun, Magdi Awadalla Mohamed, Ahmed Hassan Fahal

**Affiliations:** 1 The Mycetoma Research Centre, University of Khartoum, Khartoum, Sudan; 2 Department of Pharmaceutical Chemistry, Faculty of Pharmacy, University of Khartoum, Khartoum, Sudan; 3 Department of Pharmaceutical Chemistry, College of Pharmacy, Jouf University, Jouf, KSA; Faculty of Science, Ain Shams University (ASU), EGYPT

## Introduction

Mycetoma is a WHO recognised neglected tropical disease that is a subcutaneous chronic granulomatous progressively morbid inflammatory disease [1]. It frequently affects young adults and children in remote rural areas. It most commonly affects field laborers and herdsmen who are in direct contact with the soil. Hence, the most common site of infection is the foot, and the hand ranks second. Less frequently, other parts of the body may also be infected [2].

The disease can either be caused by true fungi, so called eumycetoma, or by certain bacteria, so called actinomycetoma, and the common causative organisms are *Madurella mycetomatis* and *Nocardia brasiliensis*, respectively [3,4]. These organisms are thought to be present in the soil, thorns, or animal dunk, and they are probably implanted into the host subcutaneous tissue through a breach in the skin as a result of minor trauma [5].

**Fig 1:**
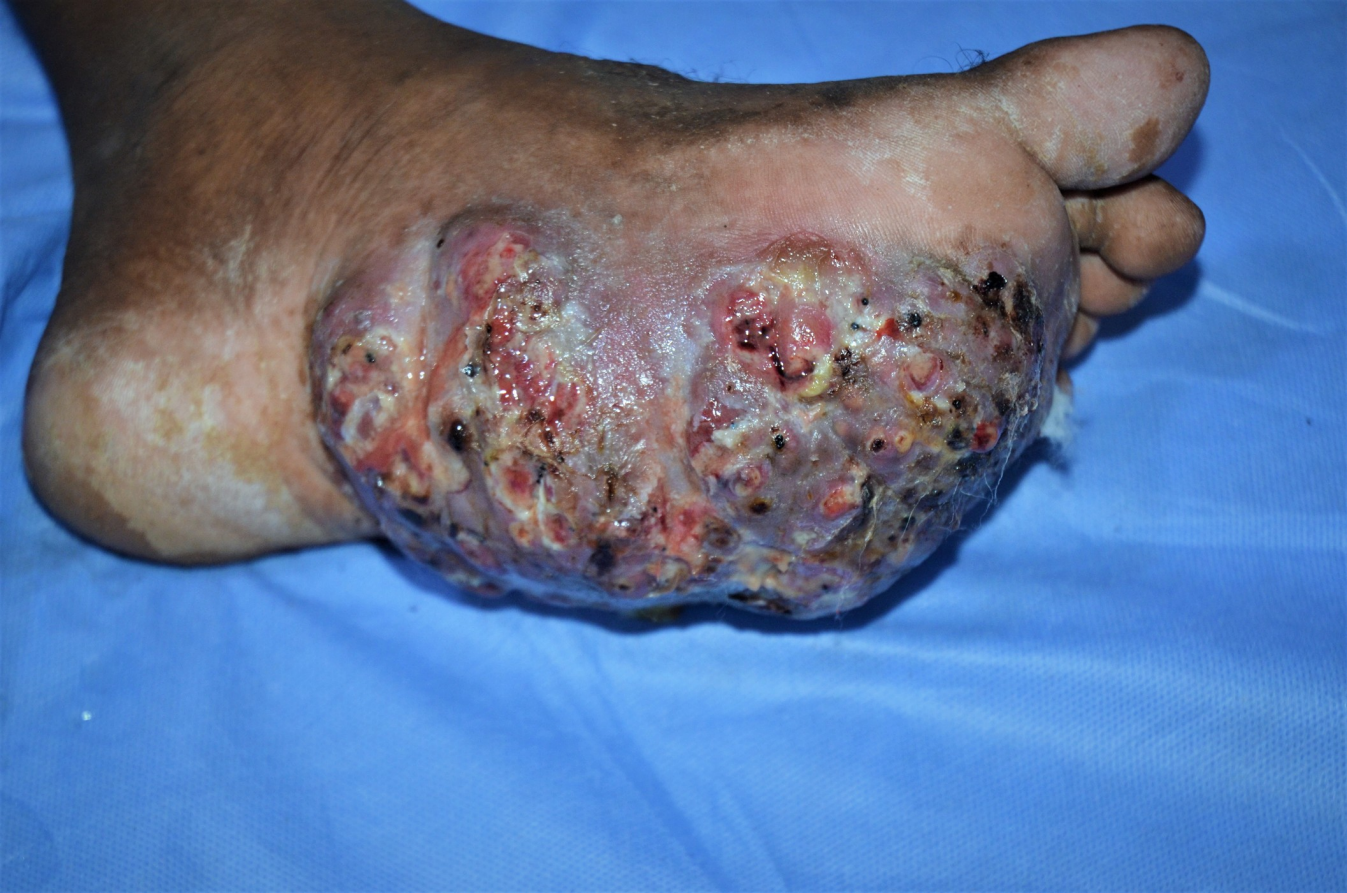
A massive eumycetoma lesion with multiple discharging sinuses and black grains.

Mycetoma, irrespective of the aetiological agent, presents as a slowly progressive, painless, subcutaneous swelling. Multiple secondary nodules then evolve that may suppurate and drain through multiple sinuses tracts. The sinuses usually discharge grains containing colonies of the causative organism, and they are considered as a unique characteristic of the disease (Fig 1) [6,7].

Actinomycetoma is relatively more responsive to medical treatment, which depends on the site, the severity of the disease, and the causative organisms, with a cure rate of up to 90% [8]. In contrast, treatment of eumycetoma is challenging and problematic, of which most cases do not respond to medical therapy alone and require alongside surgical intervention. In general, the treatment outcome of eumycetoma is suboptimal and unsatisfactory in many patients [9,10].

The disease then spreads to involve the skin, subcutaneous tissue, deep structures, and bone, resulting in destruction, deformity, loss of function, and, occasionally, mortality [7].

This Review highlights the currently available treatment options for eumycetoma caused by *M*. *mycetomatis* and their shortcomings, possible factors contributing to treatment failure, and prospects for achieving better treatment outcomes.

## Diagnosis of eumycetoma

The appropriate treatment of mycetoma depends on precise identification of the causative agent to the species level and the disease extent. For the latter, many imaging techniques are required, and include conventional X-ray radiography, ultrasonography, computed tomography (CT), and magnetic resonance imaging (MRI) [11–15]. Molecular techniques such as species-specific polymerase chain reaction (PCR), serodiagnosis as ELISA, and counter-immunoelectrophoresis as well as the classical grain culture and surgical biopsy histopathological examination are all needed to achieve accurate organism identification [11,16–18]. These techniques are not only important for diagnosis, but they also aid in treatment follow-up and assessment of cure. However, most of these techniques are invasive, expensive, of low sensitivity and specificity, and not available in endemic regions, and, hence, there is a desperate need for developing simple tests and point of care diagnostic tools.

## Treatment of eumycetoma

Despite centuries of recognition, the treatment of eumycetoma remains challenging, difficult, and disappointing. Until now, there are no definite treatment guidelines or protocols. Therefore, the treatment is based on personal experiences or a few published case reports and case series [9,10].

**Fig 2:**
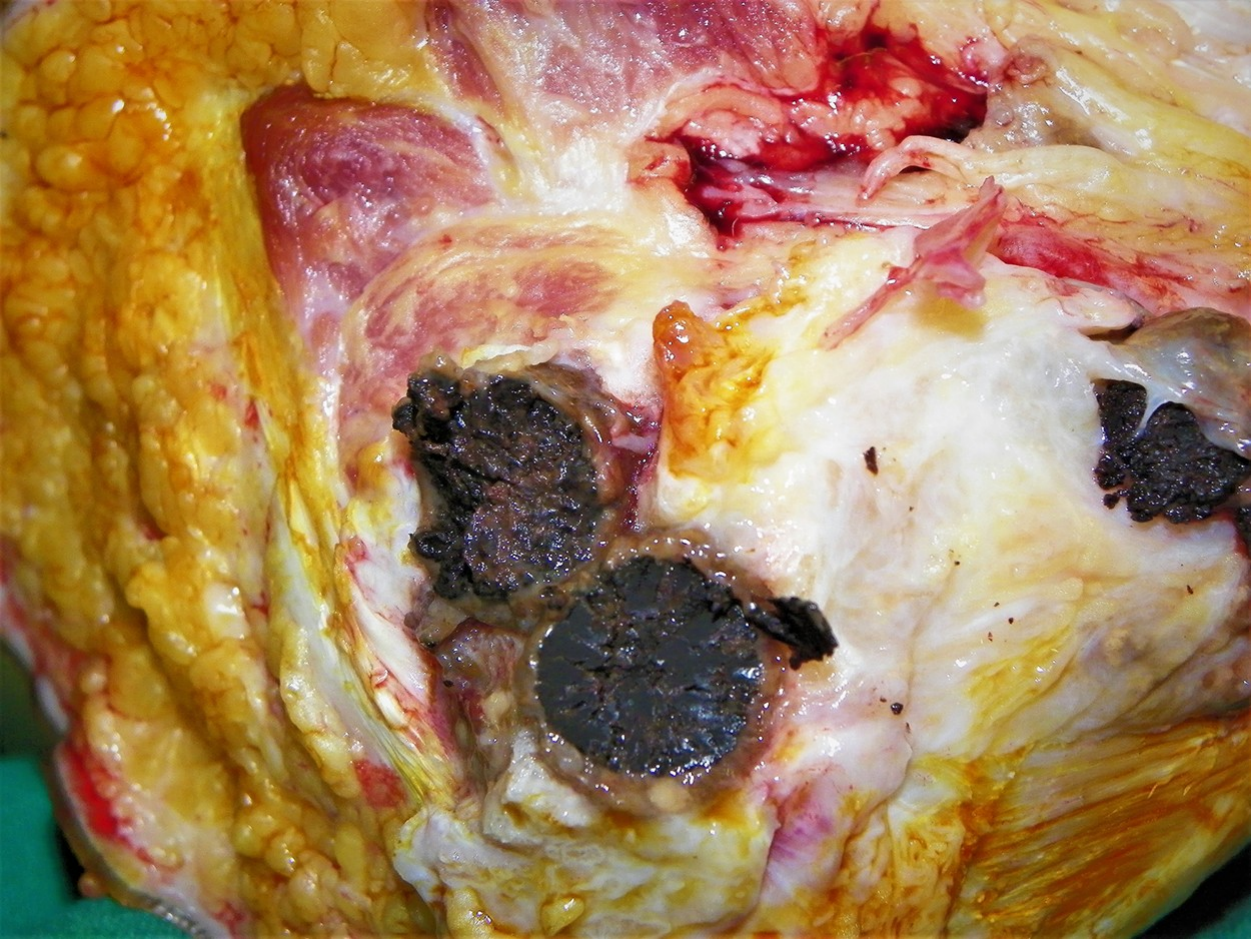
Massive postoperative recurrence after adequate itraconazole treatment for one year.

Currently, the treatment starts with preoperative antifungal treatment for six months, which continues postoperatively for at least six months [9,10]. Surgical intervention is usually in the form of adequate wide local excision, repeated aggressive debulking and debridement, or amputation in advanced disease. It aims to reduce the lesion size for better response to medical treatment or complete removal of the bacterial infected lesion [19]. The adjunct antifungal treatment is always necessary to localise the disease by forming a thick capsule around the lesion which facilitates the surgical excision that may reduce the recurrence rate (Fig 2) [19]. However, the literature showed some reported cases that showed clinical improvement with medical treatment only without surgical intervention [20–25]. There is no report of eumycetoma spontaneous cure.

## Currently used drugs for eumycetoma

Various classes of antifungal drugs have been used in the treatment of eumycetoma caused *M*. *mycetomatis* over the years, and that included the azoles, amphotericin B, and terbinafine Table 1.

**Table 1:** Reports on antifungals used for mycetoma treatment.

Drug	Study	No. of Patients	Dosage	Duration	Clinical Outcome	Principal Chronic Adverse Effect
**Liposomal amphotericin B**	Welsh et al. (2014) [10	4	3 mg/kg	6 weeks	No improvement	Nephrotoxicity
	Sampaio FMS et al (2017) [27]	1	1 mg/kg	Not specified	No improvement	
**Terbinafine**	N’Diaye et al (2006) [25]	10	1,000 mg /day	6–12 months	Responses ranged from cure to no improvement or even deterioration	Hepatotoxicity
	Seck et al (2019) [29]	1	750 mg/day	8 months	Death	
**Ketoconazole**	Venugopal et al (1993) [20]	4	400 mg/day	8–12 months	Good improvement in 3 patients, while one had only slight improvement	Life-threatening hepatotoxicity
	Mahgoub et al (1984) [32]	13	100–400 mg/day	3–36 months	Responses ranged from cure to no improvement or even deterioration	
**Itraconazole**	Fahal et al (2011) [34]	13	200–400 mg/day	12 months	Responses ranged from cure to massive recurrence	Hepatotoxicity
**Voriconazole**	Lacroix et al (2005) [22	1	400–600 mg/day	16 months	Cure	Hepatotoxicity
	Loulergue et al (2006) [23]	1	400 mg/day	12 months	Good improvement	
**Posaconazole**	Negroni et al (2005) [24]	2	800 mg/day	12 months	One patient had good improvement while the other showed no improvement	Hepatotoxicity

The toxicity and the need for hospitalization have greatly limited the use of amphotericin B for treatment of eumycetoma [26]. Liposomal amphotericin B was used in four patients at the Mycetoma Research Centre in Sudan (with a dose of 3 mg per kg) and one patient in Brazil (with a dose of 1 mg per kg). However, the clinical response was not satisfactory, and some of the patients experienced severe nephrotoxicity [10,27]. Intralesional administration of amphotericin B was reported in a case of eumycetoma caused by *Madurella grisea* in Brazil, and it resulted in a relatively good improvement [28]. However, in eumycetoma caused by *M*. *mycetomatis*, the lesions are usually multilobulated; hence, the even diffusion of the drug may not be possible. Furthermore, it is a painful procedure and may disseminate the infection.

The use of terbinafine was reported in a study in Senegal where patients were treated with 500 mg twice a day for 24 to 48 weeks that resulted in significant improvement of 80% of the patients [25]. Terbinafine use was also reported on a 13-year-old Senegalese boy with a dose of 750 mg per day. Nevertheless the boy passed away after 8 months of treatment [29]. The limited use of terbinafine could be attributed at least to its high cost and hepatotoxicity [30].

Generally, azoles remain the most commonly used class of antifungal drugs in the treatment of eumycetoma. Before it was banned in 2013, due to life-threatening hepatotoxicity [31], oral ketoconazole in a dose of 100 to 800 mg per day, was the treatment of choice [20,32,33]. It was then replaced by itraconazole in a daily dose of 200 to 400 mg per day [21,34]. Some newer azoles, such as voriconazole (400 to 600 mg per day) and posaconazole (800 mg per day), have also been employed in the management of some eumycetoma patients with good clinical outcomes [22–24]. However, their high cost compared to itraconazole might have limited their use in poor developing countries where the disease is endemic.

Itraconazole is considered as the most commonly used azole for eumycetoma treatment [10]. However, itraconazole suffers from several inadequacies which include the following.

### Suboptimal treatment outcomes and recurrence

**Fig 3:**
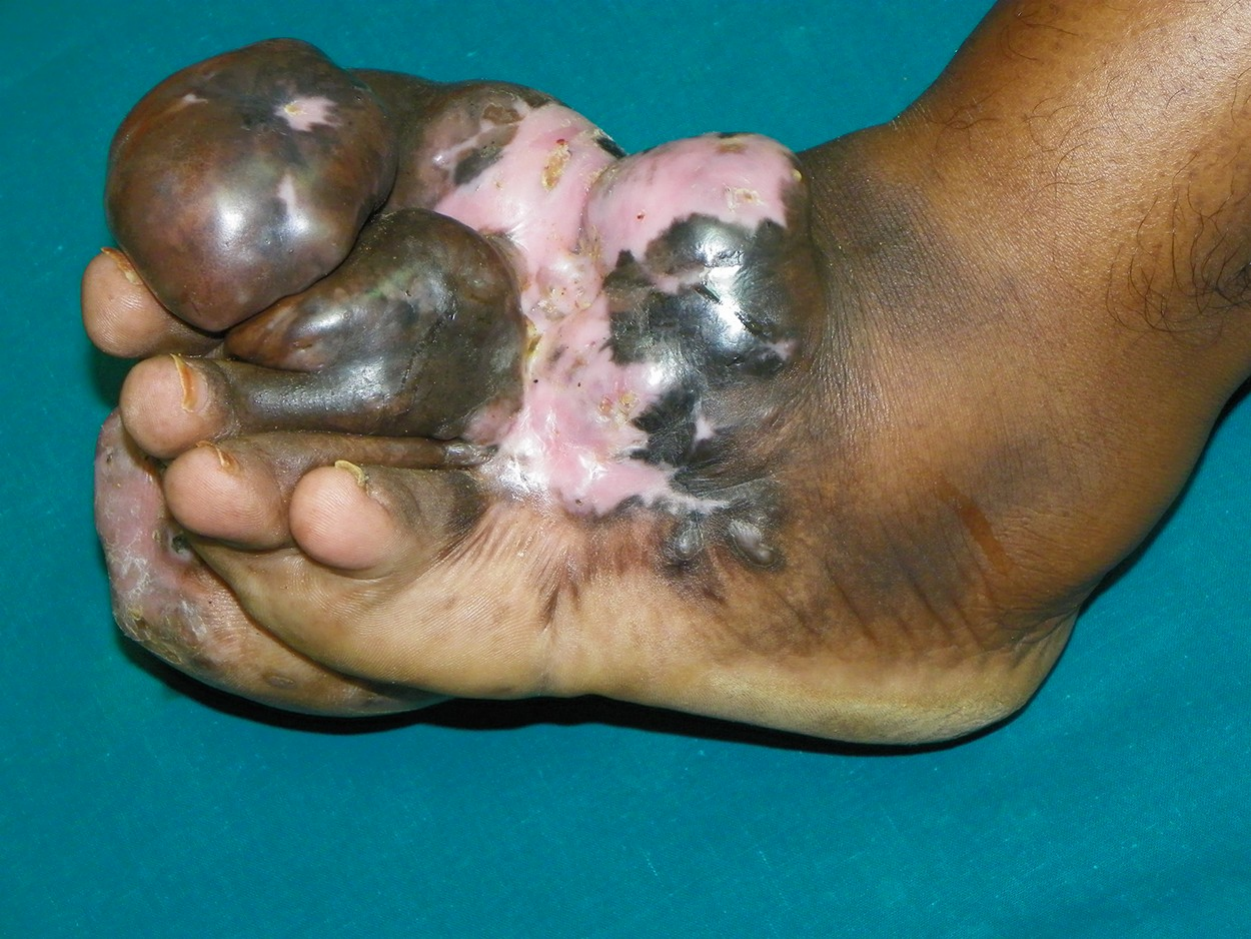
Melanin in histopathology.

The clinical response to itraconazole is often variable and is often associated with recurrence even after extended treatment periods before and after surgery (Fig 3). In one study, 13 patients were treated with itraconazole for 12 months in a dose of 400 mg per day for three months and then reduced to 200 mg per day for nine months; only one patient showed complete cure, nine patients showed partial response, and the rest three had stable disease. Later, one patient had a massive recurrence after partial cure [34]. In another larger prospective study, only 321 of 1,242 (25.9%) eumycetoma patients were cured [35]. Despite prolonged treatment with itraconazole before and after surgery, postoperative recurrence is quite common. Recurrence was reported in 276 of 1,013 patients (27.2%) treated by itraconazole accompanied by surgery at the MRC in Sudan [36].

### Prolonged treatment duration and adverse effects

Extended treatment duration with itraconazole was shown to be an important predictor for attaining higher cure rates in eumycetoma patients [35]. Thus, eumycetoma patients usually need to endure 6 months to 3 years of treatment with itraconazole [37]. This in turn greatly affects patients’ adherence and compliance and results in high follow-up dropout rates [35]. Such prolonged treatment periods also make patients more vulnerable to serious adverse effects. Like other azoles, lengthy use of itraconazole affects liver functions, ranging from transient elevations in serum transaminases to hepatoxicity and liver failure [30].

Additionally, itraconazole has negative inotropic effects on the heart and has therefore been associated with congestive heart failure [38–40]. Being an azole, itraconazole is contraindicated in pregnancy since it is embryotoxic and teratogenic in animals (Pregnancy Risk Category C) [41]. Moreover, pregnant women exposed to itraconazole were shown to have an increased risk of early fetal loss [42].

### Organism viability within the grains

Even though *M*. *mycetomatis* is highly susceptible to itraconazole in vitro [43,44], grains containing viable fungi were isolated from patients who were on prolonged treatment with itraconazole [34]. Hence, itraconazole seems to only limit the extent of the infection instead of complete eradication of the *M*. *mycetomatis’* tissue burden [45].

### Drug pharmacokinetics

The pharmacokinetic profile of itraconazole is known to have considerable interpatient variability while using the same dose of the drug [46]. This erratic variation could largely be attributed to the fact that the absorption of itraconazole form the gastrointestinal tract is greatly influenced by stomach acidity and concomitant food intake [47–50]. At best, the amount of itraconazole available for therapeutic activity represents only 0.11% of the ingested dose. This could be attributed to the fact that the absolute oral bioavailability of itraconazole is only 55% and, of that absorbed fraction, 99.8% is bound to plasma proteins and thus considered unavailable for therapeutic effects [50–52]. Consequently, this pharmacokinetic profile of itraconazole adds unnecessary cost to the patients. Furthermore, due to high protein binding itraconazole can only reach the cerebrospinal fluid in minimal amounts [50,52], this limits its therapeutic effectiveness in cerebral eumycetoma infections.

### Drug–drug interactions

Eumycetoma is a chronic medical condition and patients may develop several comorbidities during the course of their infection. This will necessitate the coadministration of drugs that might have undesirable pharmacokinetic interactions with itraconazole, at the level of absorption, distribution, metabolism, or excretion [53–55]. These interactions could result in decreased or increased plasma levels of itraconazole or coadministered drugs, thus leading to reduced efficacy or increased toxicity, respectively. For instance, administration of acid neutralising (e.g., aluminium hydroxide) or suppressing (e.g. H2-antagonists as ranitidine or proton-pump inhibitors as omeprazole) drugs lead to inadequate absorption of itraconazole [56–58]. Itraconazole is a potent inhibitor and also a substrate of Cytochrome P450 3A4 (CYP3A4), which is responsible for the metabolism of a broad range of drugs [59,60]. Consequently, itraconazole will increase plasma concentrations of CYP3A4 substrates, while inducers and inhibitors of CYP3A4 will decrease or increase itraconazole plasma concentrations, respectively. Therefore, levels of itraconazole and other coadministered drugs should be closely monitored to avoid subclinical concentrations or undesired toxic effects of both drugs (Table 2).

**Table 2:** Some drugs that could affect the metabolism of itraconazole or be affected by itraconazole coadministration.

Drugs affecting itraconazole metabolism	Drugs which metabolism is inhibited by itraconazole
*Drugs inhibiting Itraconazole metabolism*	Antihistamines (e.g., astemizole, terfenadine)
HIV protease inhibitors (e.g., ritonavir, indinavir) *	Benzodiazepine sedatives (e.g., midazolam, diazepam, triazolam, alprazolam)
Macrolide antibiotics (e.g., erythromycin and clarithromycin)	Calcium channel blockers (e.g., amlodipine, nifedipine)
*Drugs inducing itraconazole metabolism*	HIV protease inhibitors (e.g., ritonavir, indinavir) *
Anticonvulsants (e.g., phenytoin, phenobarbital, carbamazepine)	HMG-CoA reductase inhibitors (e.g., lovastatin, atorvastatin)
Antimycobacterials (e.g., rifampin, isoniazid)	Oral hypoglycaemics (e.g., glimepiride, chlorpropamide, metformin).
	Oral anticoagulants (e.g., Warfarin)
	Immunosuppressants (e.g., Cyclosporine)
	Anticancers (e.g., vincristine)
	Digoxin
	Cisapride
	Methylprednisolone
	Sildenafil citrate
	Quinidine

* Concomitant use of itraconazole with protease inhibitors may result in a dual interaction that leads to changes in plasma concentrations of both drugs.

### Combination therapy

Most invasive fungal infections are difficult to treat with antifungal monotherapy. Thus, combining antifungal drugs seems to be a promising approach to achieve synergistic effects that could improve overall efficacy and decrease the duration of treatment, toxicity, and possibly resistance [61].

Antifungal combination therapy can produce synergy via several mechanisms. One mechanism could involve the inhibition of different stages of one biochemical pathway. Such synergy could be seen in the combination of azoles and terbinafine, in which they affect the integrity of the fungal cell membrane by targeting ergosterol biosynthesis at various levels [62]. The use of such combinations has been reported for eumycetoma caused by *M*. *mycetomatis*. In India, a patient was successfully treated using a combination of itraconazole (400 mg per day) and terbinafine (250 mg per day) [63]. In another case series, two patients were treated with a combination of voriconazole (400 to 700 mg per day) or posaconazole (800 mg per day) with terbinafine (dose not specified). One patient did not respond to treatment, while the other showed very good clinical improvement [64].

Synergy could also be achieved by combining azoles with flucytosine, in which azole damage the fungal cell membrane, thus enhancing the penetration of flucytosine to its target, where it inhibits the synthesis of both DNA and RNA [62,65]. The use of posaconazole (800 mg per day) combined with flucytosine (80 mg per kg per day) was also reported to produce good clinical improvement in eumycetoma patients [64,66].

The treatment of eumycetoma caused by *M*. *mycetomatis* is often complicated by the development of bacterial coinfections, most commonly by *Staphylococcus aureus* [67]. Combining antibacterial drugs such as an amoxicillin–clavulanic acid (2 g per day) with the regular antifungal regimen helps in eradicating the bacterial coinfection and, hence, improving the overall clinical outcome of the patients [68]. The use of such combinations has been reported in the literature but without a clear rationale for their administration because no bacterial coinfections were described. One eumycetoma patient showed good clinical response upon treatment with a combination of intravenous trimethoprim–sulfamethoxazole and liposomal amphotericin B (doses not specified) then a combination of oral trimethoprim–sulfamethoxazole (320 mg per day) with posaconazole (800 mg per day) and ciprofloxacin (1 Gram per day) [69]. Similarly, the combination of itraconazole with trimethoprim–sulfamethoxazole (doses not specified) resulted in good improvement in two patients in Brazil. The authors suggested that sulfamethoxazole–trimethoprim might have some activity against the causative fungi [70].

Eumycetoma is usually associated with intense inflammatory reactions produced by the host tissue [71], which are proposed to play an important role in the pathogenesis of the disease [66]. Hence, combining antiinflammatory drugs with antifungal therapy could help in improving the clinical outcomes of eumycetoma patients. Addition of the nonsteroidal antiinflammatory drug, diclofenac (100 mg per day), to a combination of posaconazole (800 mg per day) and flucytosine (80 mg per kg per day), resulted in complete normalisation of the clinical picture within two months of a patient who had refractory mycetoma for over 20 years [66]. In Brazil, combining oral prednisolone with antifungals and sulfamethoxazole plus trimethoprim was also reported to enhance the clinical improvement cure rates of patients without causing additional side effects [72].

### Possible barriers to effective treatment

In vitro, *M*. *mycetomatis* is susceptible to various classes of antifungals, yet the clinical outcomes of these agents are unsatisfactory [44,73–76]. Many co-operating factors might contribute to these poor clinical outcomes, such as:

### Grains melanin

**Fig 4:**
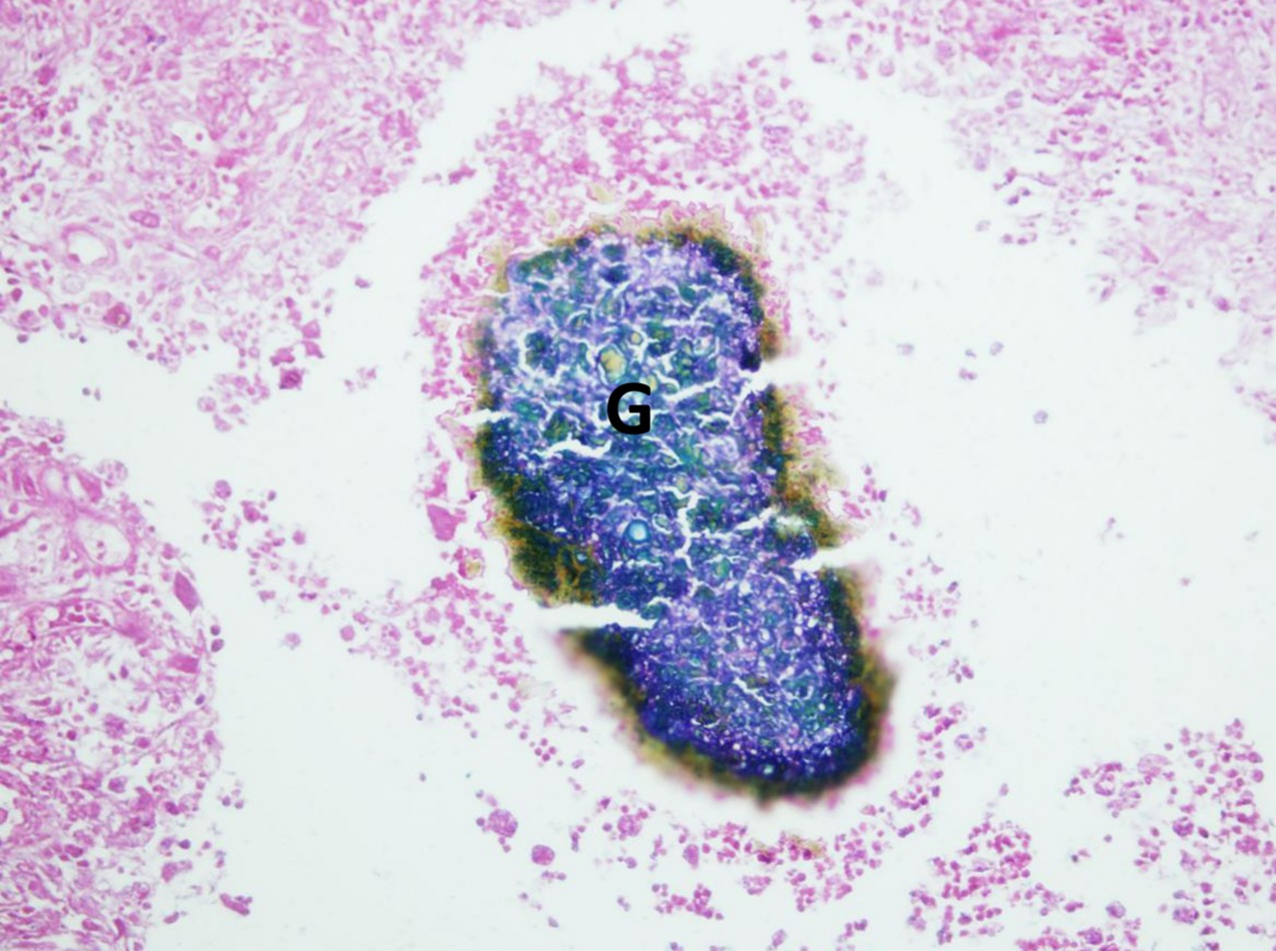
Massive thick capsule around the eumycetoma lesions.

The black colour *of M*. *mycetomatis* grains is due to the fungi ability to produces two types of melanin: pyo-melanin (soluble and secreted by the fungus) and dihydroxynaphthalene (DHN)-melanin (solid, insoluble, and, usually, bound to the cell wall) Fig 4 [11]. The latter melanin was shown to reduce the in vitro efficacy of itraconazole and ketoconazole by 16- and 32-folds, respectively [77]. This was explained by the fact that DHN-melanin hinders the accessibility of these drugs to the fungal mycelia [77,78]. Cell-mediated immunity plays a major adjunct role to drugs in the control and eradication of fungal infections [79]. DHN-melanin was found to protect *M*. *mycetomatis* in vitro from the killing effects of permanganate: one of the strongest known oxidants. Hence, inside the host, this melanin may act as a scavenger for immune oxidants, such as nitric oxide produced against the fungal invasion [77]. Interestingly, fungi have been shown to increase the production of DHN-melanin when challenged with itraconazole, possibly conferring additional protection against the drug [80].

### The collagen

**Fig 5:**
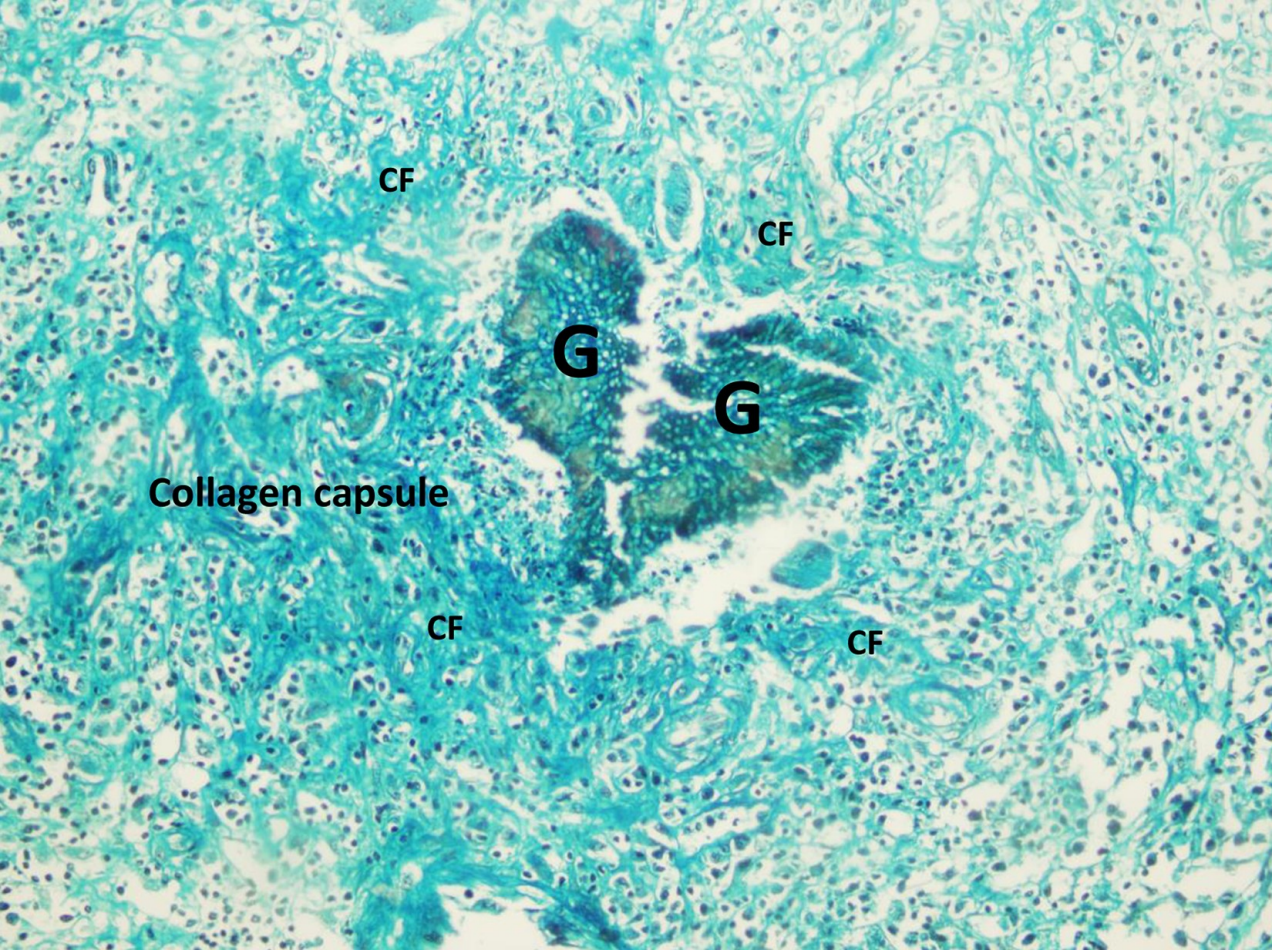
Photomicroscopy showing *M*. *mycetomatis* grains well encapsulated with excessive collagen following treatment with itraconazole (hematoxylin–eosin X 400).

Following prolonged treatment with itraconazole or ketoconazole, *M*. *mycetomatis* grains are usually found to be encapsulated with excessive collagen (Fig 5). This collagen accumulation was found to be associated with elevated levels of active matrix metalloproteinases-9 (MMP-9) in eumycetoma patients [81] that probably disrupts the equilibrium of the extracellular matrix (ECM) synthesis and degradation. Such dense collagen networks around the fungal lesion might localize the infection, though it has also been suggested that it might hinder drug accessibility and, hence, diminish the response to antifungal treatment [81]. Such effects of collagen on penetration have been reported in macromolecules; however, similar effects on micromolecules such as antifungal agents need further investigation [82,83].

### The patients’ late presentation

**Fig 6:**
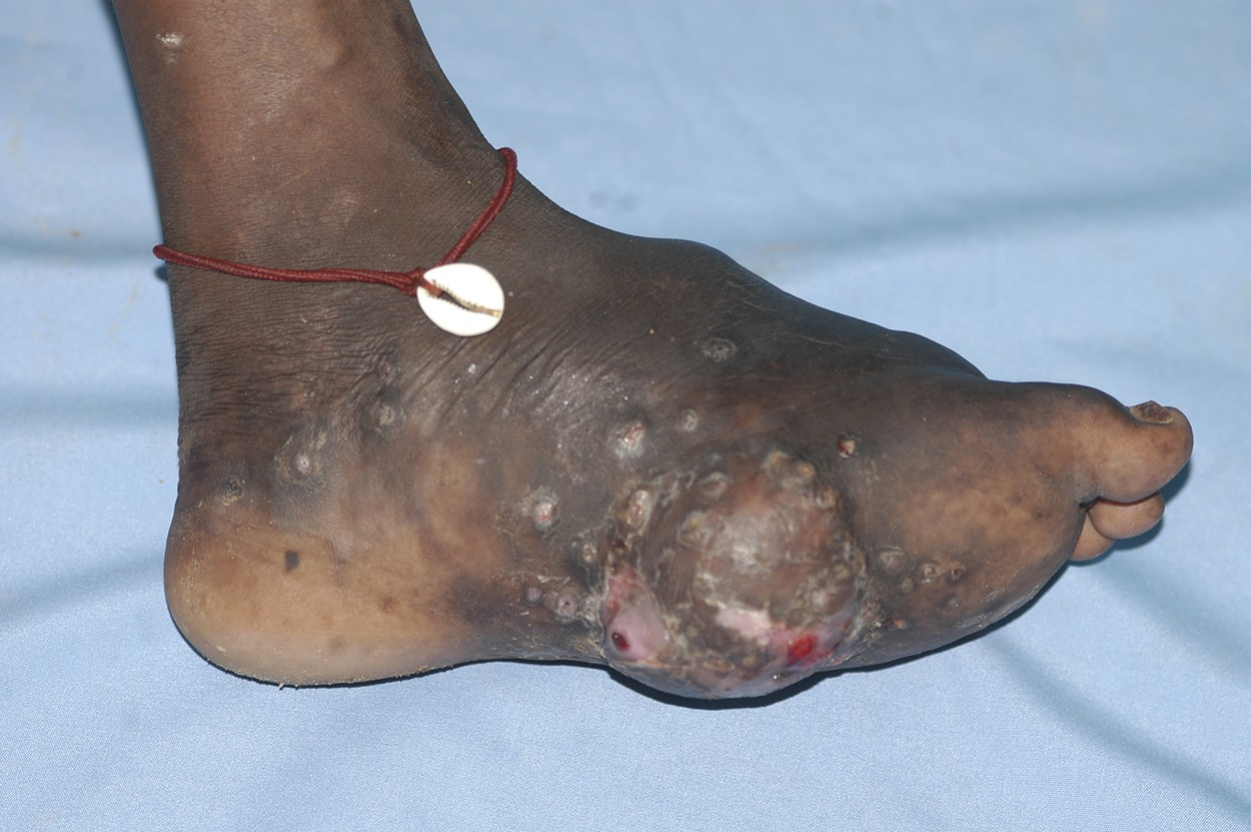
The use of traditional medicine for mycetoma.

A major problem with eumycetoma patients is the fact that they tend to present to treatment at late stages with advanced disease (the median duration of the disease at presentation is three years) [32]. This long disease duration seems to be an important predictor of poor treatment outcomes [35]. This late presentation of eumycetoma patients may be attributed to the substantial lack of health education and health facilities in rural areas where eumycetoma is endemic. Furthermore, the high coast and far away access to treatment combined with the patients’ low socioeconomic status led them to first seek other treatment alternatives, such as herbal and traditional medicine. Approximately 42.4% of eumycetoma patients have used herbal medicine during the course of their disease [84]. Herbs such as *Moringa oleifera*, *Acacia nilotica*, *Citrullus colocynthis*, and *Cuminum cyminum* were commonly used either alone or in combination with other herbs [84]. Some patients also seek traditional and religious healing techniques such as cautery, charms, amulets, hijabs, erasure (mihaya), and incantations (ruqia) (Fig 6) [85]. These alternative treatments are usually not only ineffective in treating eumycetoma but also lead to serious complications such as skin burns, necrosis, and, most importantly, secondary bacterial infections [84].

### Recommendation for improving currently available treatment

As has been stated, treatment of eumycetoma suffers from several shortcomings, most importantly the limited treatment alternatives, which are associated with low cure rates and great variability in response among patients. As have been mentioned previously, combining antifungal drugs for the treatment of eumycetoma caused by *M*. *mycetomatis* have shown some promising outcomes. However, these outcomes are limited to only a few case reports [63,64,66]. That is why there is an urgent need for proper and controlled clinical studies in larger numbers of patients to determine the most effective combination of drugs, their doses, and duration of treatment. Furthermore, the outcomes of these case reports do not coincide with in vitro and in vivo findings (using *M*. *mycetomatis*-infected *Galleria mellonella* larvae), in which drug combinations did not result in synergy nor improved the therapeutic response [86,87]. Developing a three-dimensional (organoid) culture system for *M*. *mycetomatis* might aid in obtaining a better reflection of the host–pathogen complex biological interactions [88] and, hence, a more accurate prediction of the fungi’s response to drugs and their combinations [89].

The variable and unsatisfactory clinical response of eumycetoma patients to itraconazole could partially be attributed to its poor pharmacokinetic profile. Maintaining effective and safe serum levels of itraconazole in patients could be achieved via therapeutic drug monitoring (TDM) techniques such as high-performance liquid chromatography (HPLC) and mass spectrometry (MS) [90]. Furthermore, enhancing the oral bioavailability of itraconazole could be a good approach to enhance its therapeutic efficacy, while reducing treatment cost. Many pharmaceutical technologies have been developed over the years to enhance the oral bioavailability of itraconazole. For example, an oral solution of itraconazole was developed via incorporating it with a cyclodextrin vehicle, which resulted in a 37% higher oral bioavailability compared to itraconazole capsule in healthy volunteers [91,92]. Furthermore, the absorption of itraconazole from this oral solution is enhanced when taken on an empty stomach [91]. Hence, using such formulations in the management of eumycetoma might improve the clinical outcome of the disease.

As have been mentioned previously, the late presentation of eumycetoma patients is a major hurdle in their treatment. Thus, implementing vigorous health education programs in mycetoma endemic areas could be a possible solution. These educational programs should also embrace the traditional healers as they are greatly trusted by the locals and could help in detecting early cases. Once enrolled in treatment, patients should be counselled on the potential drug–drug and drug–food interactions of itraconazole, so as to maximize the effectiveness of the drug and minimise its potentially toxic effects.

Eumycetoma results in serious disfigurement, scarring, and disability. Hence, patients are often stigmatized in their communities. This could lead to patients being reluctant to seek medical treatment once they notice their disease. Therefore, providing psychological support and occupational rehabilitation for these patients could improve their adherence to treatment and hence improve their treatment outcome.

### Drug discovery for eumycetoma

As mentioned in the previous parts of the Review, the success rate of the currently available treatment options for eumycetoma caused by *M*. *mycetomatis* is minimal. Accordingly, there is a desperate need for finding new medicines to address the unmet clinical need in the field of eumycetoma management.

Drug discovery for eumycetoma can take place by several means. Herein, two main ways will be discussed. Firstly, the drug repurposing approach and secondly the de novo drug discovery. In this issue, the term “de novo drug discovery” represents the discovery of novel treatments through the regular drug discovery pipeline, which involves the screening of chemical compounds, followed by in vitro, in vivo testing then preclinical and clinical evaluation [93]. On the other hand, drug repurposing refers to the use of approved medication for indications other than the one that it was originally developed for [94]. Thus, drug repurposing could aid in finding novel medicines, while dramatically cutting down expenses and shortening the drug discovery process by relying on existing safety and pharmacokinetic profiles of drugs that are already in the market [94,95].

### Drug repurposing for eumycetoma

Drug repurposing became an attractive approach for finding new treatments for diseases like eumycetoma because these diseases occur primarily and almost solely in poor communities. Hence, these diseases do not represent an attractive investment for pharmaceutical companies, as their profits are not considered satisfactory enough to compensate for the cost of the de novo drug discovery [96].

Among the examples of drug repurposing for eumycetoma, treatment is fosravuconazole. The starting point for the application of fosravuconazole for treating eumycetoma was ravuconazole. It is a newly developed broad-spectrum triazole that was initially developed for the treatment of Chagas disease. In vitro studies showed that ravuconazole is active against *Madurella mycetomatis* [74]. Nevertheless, ravuconazole is too expensive to be applied directly in the treatment of eumycetoma, a disease that is almost restricted to underprivileged communities. Fortunately, Eisai, a Japanese pharmaceutical company, developed a more affordable prodrug of ravuconazole called fosravuconazole that is presently being clinically assessed in the first double-blind clinical trial on eumycetoma patients at the MRC in Sudan [97]. This trial hopes to deliver effective and affordable treatment for eumycetoma.

### The de novo drug discovery

The chief drawback of the de novo drug discovery is the fact that it is a long, time-consuming, and financially demanding journey [98]. Nevertheless, there are still some initiatives such as the Drugs for Neglected Diseases initiative (DNDi) that support the discovery of treatments for neglected diseases, such as eumycetoma, through this path [99]. Among the DNDi moves towards finding new efficacious medications for eumycetoma was to provide chemical entities for screening against *M*. *mycetomatis*. In one of the studies, the in vitro and in vivo screening of more than 800 different compounds resulted in the identification of several new hits that could potentially be developed into effective drugs for eumycetoma caused by *M*. *mycetomatis* [100]. These previous findings were the starting point of the Mycetoma Open Source project (MycetOS) in 2018 [101], which focuses on discovering novel treatments for eumycetoma through an Open Pharma approach [102]. Through an open-access database that is publicly driven, the project aims at the discovery of new drugs and the optimization of available leads for management of eumycetoma caused by *M*. *mycetomatis*. Thus, MycetOS does not belong to any specific person or organization. It, rather, belongs to everyone who is willing to participate.
